# Population dynamics of an *Escherichia coli* ST131 lineage during recurrent urinary tract infection

**DOI:** 10.1038/s41467-019-11571-5

**Published:** 2019-08-13

**Authors:** Brian M. Forde, Leah W. Roberts, Minh-Duy Phan, Kate M. Peters, Brittany A. Fleming, Colin W. Russell, Sara M. Lenherr, Jeremy B. Myers, Adam P. Barker, Mark A. Fisher, Teik-Min Chong, Wai-Fong Yin, Kok-Gan Chan, Mark A. Schembri, Matthew A. Mulvey, Scott A. Beatson

**Affiliations:** 1 School of Chemistry and Molecular Biosciences, The University of Queensland, Brisbane, 4072 Queensland Australia; 2 Australian Infectious Diseases Research Centre, The University of Queensland, Brisbane, 4072 Queensland Australia; 3 Australian Centre for Ecogenomics, The University of Queensland, Brisbane, 4072 Queensland Australia; 4 Division of Microbiology and Immunology, Department of Pathology, University of Utah School of Medicine, Salt Lake City, 84132 UT USA; 5 Genitourinary Injury and Reconstructive Urology, Department of Surgery, University of Utah, Salt Lake City, 84132 UT USA; 6 ARUP Laboratories and Department of Pathology, University of Utah, Salt Lake City, 84112 UT USA; 7 Division of Genetics and Molecular Biology, Institute of Biological Sciences, Faculty of Science, University of Malaya, Kuala Lumpur, 50603 Malaysia; 8 International Genome Centre, Jiangsu University, Zhenjiang, 212013 China

**Keywords:** Urinary tract infection, Comparative genomics, Clinical microbiology

## Abstract

Recurrent urinary tract infections (rUTIs) are extremely common, with ~ 25% of all women experiencing a recurrence within 1 year of their original infection. *Escherichia coli* ST131 is a globally dominant multidrug resistant clone associated with high rates of rUTI. Here, we show the dynamics of an ST131 population over a 5-year period from one elderly woman with rUTI since the 1970s. Using whole genome sequencing, we identify an indigenous clonal lineage (P1A) linked to rUTI and persistence in the fecal flora, providing compelling evidence of an intestinal reservoir of rUTI. We also show that the P1A lineage possesses substantial plasmid diversity, resulting in the coexistence of antibiotic resistant and sensitive intestinal isolates despite frequent treatment. Our longitudinal study provides a unique comprehensive genomic analysis of a clonal lineage within a single individual and suggests a population-wide resistance mechanism enabling rapid adaptation to fluctuating antibiotic exposure.

## Abstract (web summary)

Recurrent urinary tract infections occur in ~ 25% of women. Here, Beatson and colleagues use whole genome sequencing to track the dynamics of an *E. coli* ST131 clone in a single patient over a 5-year period. This study provides unique insights into pathogen evolution during recurrent urinary infection.

## Introduction

Uropathogenic *Escherichia coli* (UPEC) are the primary cause of urinary tract infection (UTI), being responsible of ~80% of all cases^1^. UPEC strains largely belong to the *E. coli* phylogenetic groups B2 or D and are often clonal, with the most common sequence types (STs) isolated worldwide being ST69, ST73, ST95 and ST131^2^. The recently emerged and globally disseminated ST131 clone is a major contributor to hospital- and community-acquired UTI^3,4^, as well as bloodstream infections^5^ and infections in companion animals and poultry^6^. Originally identified in 2008, ST131 is associated with the worldwide spread of the CTX-M-15 extended spectrum β-lactamase (ESBL) resistance gene^7,8^. Most ST131 strains are now strongly associated with multidrug resistance (MDR)^9,10^, including resistance to fluoroquinolones^11^. Recent reports have also identified strains that are resistant to last-line carbapenems^12,13^. Overall, these developments have limited treatment options for UTIs and bloodstream infections caused by ST131 strains and resulted in an increased frequency of recurrent infection.

Several large epidemiology studies have delineated the genomic phylogeny of ST131 into three major clades: clade A (being the most divergent and represented by the reference strain SE15), clade B (associated with animal to human transmission)^14^, and clade C (represented by the reference strain EC958)^15,16^. Clade C (also known as *H*30) represents the largest clade of ST131 and comprises two sub-lineages, C1 (or *H*30R) and C2 (or *H*30Rx), both of which are resistant to fluoroquinolones. ST131 strains containing the CTX-M-15 ESBL allele, the most widespread CTX-M ESBL^3^, represent a dominant proportion of clade C2^15,16^. Recent phylogenomic studies have defined the evolutionary history of resistance to fluoroquinolones in clade C^17,18^ and revealed the genetic steps that led to the emergence of this lineage^18^. While the core genome of ST131 is highly conserved (comprising >3150 genes and 3,186,979 bp^15^), there is variation in the accessory genome, resulting in differences in virulence gene repertoire^15^ and plasmid content^19^.

Recurrent UTIs (rUTI) are symptomatic infections caused by the re-introduction of bacteria into the urinary tract or the resurgence (recrudescence) of bacterial reservoirs following clinical resolution of a previous UTI^20^. rUTIs are extremely common among women; ~50% of women with UTI experience a recurrence within 1 year, ~25% of whom will experience a recurrence within 6 months of the initial infection^21–24^. The frequency of rUTI in older women is not well documented, but it is estimated that 10–15% of women over the age of 60 years experience multiple rUTI episodes^23^. Although persistent infections can occur, in many cases rUTIs result from reinfection by the same organism^20,25,26^. In UTI caused by UPEC, >60% of recurrences can be attributed to the original infecting strain^25^. These infecting strains can persist in the gut after elimination from the bladder and subsequently recolonise the bladder and cause another UTI^25^. Indeed, the prototype ST131 strain EC958 was recently shown to efficiently colonise the mouse gut and persist long term^27^.

Here we characterise the evolution of ST131 strains associated with chronic rUTI in a single individual (Patient #1) over a 5-year period. Our analysis of both long- and short-read genome sequencing data identifies a highly dynamic and complex intestinal population that likely serves as a reservoir for rUTI in this individual. Furthermore, we provide evidence demonstrating the transfer of antibiotic resistance genes and plasmids within a clonal population of ST131 over the course of several years.

## Results

### Clinical case study

Patient #1 is a Caucasian female who first presented to the Urology Clinic at the University of Utah Hospital in 2011 at age 74 years. She has a pertinent past medical history of lifelong irritable bowel symptoms and colonic diverticulosis and has been taking amitriptyline for nausea since the early 1980s. Patient #1 also uses the angiotensin-converting-enzyme inhibitor Lisinopril (20 mg daily), the beta blocker Atenolol (50 mg daily), and the diuretic Hydrochlorothiazide (25 mg daily) to control high blood pressure and the non-steroidal anti-inflammatory drug Meloxicam (15 mg as needed) for back pain. She regularly takes vitamin pills, including iron supplements. Patient #1 experiences frequent UTIs, which began soon after a vaginal hysterectomy in 1971. Her UTIs are accompanied by symptoms of dysuria and urinary frequency/urgency, fatigue, and bladder pain, all of which usually resolve with administration of culture-specific antibiotic treatments. In 2010, outside hospital providers became concerned about a possible colovesical fistula based on the patient’s history of recurrent UTIs and the close proximity of her colon to the bladder, as revealed by computed tomographic scan imaging. Patient #1 subsequently underwent resection of 12 inches of colon, and a flap of omentum was placed between the colon and bladder wall. This intervention did not reduce her UTI frequency.

Over the years, Patient #1 was seen by multiple healthcare providers who prescribed a variety of antibiotics (Supplementary Table 1), including beta-lactams (amoxicillin, carbapenems, third- and fourth-generation cephalosporins), nitrofurantoin, gentamicin, trimethoprim/sulfamethoxazole, fluoroquinolones (ciprofloxacin, levofloxacin), and the prodrug pyrazinamide. In 2012, *E. coli* isolated from a clean-catch urine sample provided by Patient #1 was found by PCR to be from the ST131 clone, which we designated as strain U12A. Antibiograms indicated that strain U12A was resistant to multiple antibiotics, including the beta-lactam class antibiotics ampicillin and cefazolin, the aminoglycosides gentamicin and tobramycin, trimethoprim/sulfamethoxazole and the fluoroquinolones ciprofloxacin, levofloxacin and moxifloxacin (Supplementary Table 2). Patient #1 was subsequently treated with a 21-day course of the carbapenem antibiotic ertapenem and remained free of symptomatic UTIs for about 9 months. The patient reports that this was the longest period that she had gone without enduring a UTI in close to 40 years. Since 2013, Patient #1 has continued to experience frequent rUTIs, despite receiving culture-specific antibiotic treatments that provide at best only temporary relief.

### Phylogeny of Patient #1 ST131 isolates

Following the culture of U12A in early 2012, four additional *E. coli* urine isolates were collected from Patient #1 over a 4-year period: U13A isolated in June 2013, U14A in February 2014, U15A in March 2015, and U15B in September 2015. All five urine isolates were determined to be ST131 by PCR and subsequent genome sequencing, and all had similar, though distinct, patterns of resistance to multiple antibiotics (Table 1; Supplementary Table 2).

**Table 1:** Antibiotic resistance profiles of P1A isolates

Gene	Resistance	Context	U12A	U13A	U14A	U15A	U15B
*dfrA17*	Trimethoprim	IncF/IncI plasmid	+	+	+	+	−
*aadA5*	Streptomycin Spectinomycin	IncF/IncI plasmid	+	+	+	+	−
*sul1*	Sulfonamide	IncF/IncI plasmid	+	+	+	+	−
*sul2*	Sulfonamide	IncF/IncI plasmid	+	+	+	+	−
*strA*	Streptomycin	IncF/IncI plasmid	+	+	+	+	−
*strB*	Streptomycin	IncF/IncI plasmid	+	+	+	+	−
*tetA*	Tetracycline	IncF/IncI plasmid	+	+	+	+	−
*aac(3)-IId*	Gentamicin	IncF/IncI plasmid	+	−	−	+	−
*bla*_TEM-1B_	Ampicillin	IncF plasmid	+	+	+	−	−
*mph(A)*	Macrolide		+	+	+	+	−
*ampC*	Cephalosporins	Chromosome	+	+	+	+	+
*gyrA* (S83L, D87N)	Fluoroquinolones	Chromosome	+	+	+	+	+
*parC* (S80I, E84V)	Fluoroquinolones	Chromosome	+	+	+	+	+

**Fig. 1:**
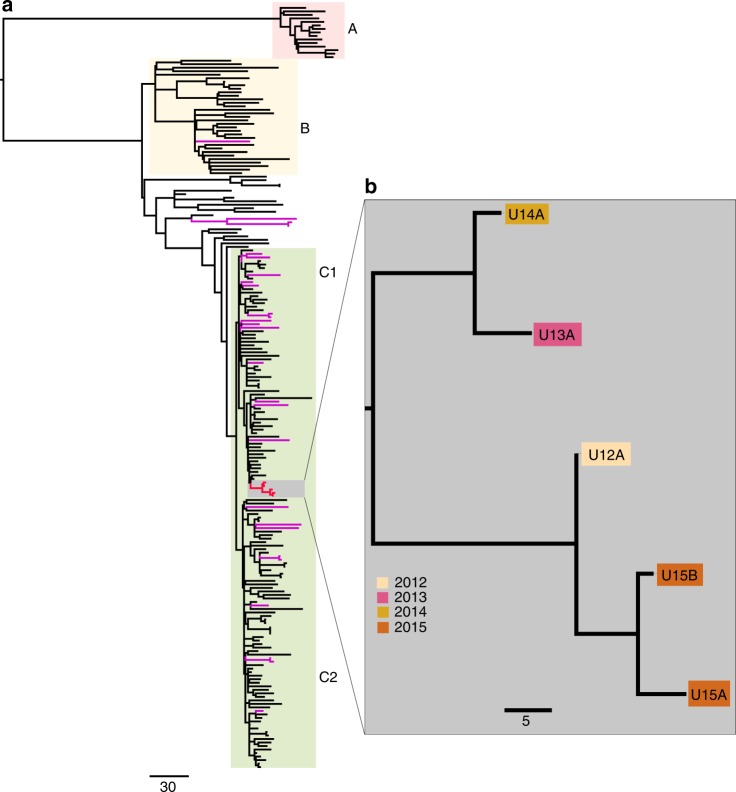
Global representation of the ST131 phylogeny. **a** Maximum-likelihood phylogenetic tree built using 4286 single-nucleotide polymorphisms (SNPs) relative to *E. coli* EC958. Patient #1 isolates are highlighted in red and the additional Utah ST131 isolates in purple. The major ST131 phylogenetic clades are indicated: A = red, B = yellow, C1 and C2 = green. **b** Zoom-in depicting a maximum-likelihood phylogenetic tree of the 5 Patient #1 ST131 isolates constructed using 26 non-recombinant core genome SNPs. Isolates are coloured based on their data of isolation as indicated in the legend. The scale bar indicates branch length in number of SNPs. Phylogenetic trees were visualised using Figtree (http://tree.bio.ed.ac.uk/software/figtree/)

**Fig. 2:**
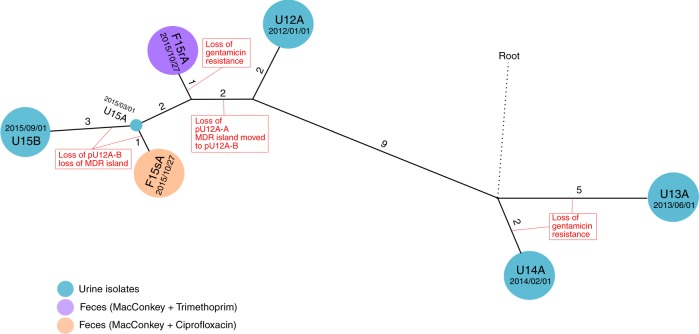
Phylogeny of Patient #1 P1A lineage. Evolutionary relationship of urine and faecal ST131 isolates from Patient #1 constructed using 37 core genome single-nucleotide polymorphisms (SNPs). Isolates are represented by the spherical nodes and coloured as per the legend. Branches connecting individual nodes represent the core genome SNP distance between isolates and are shown to scale. The core genome SNP distance between isolates is indicated by the number adjacent to each branch

ST131 is a globally distributed UPEC clone and it has been previously shown that isolates from disparate sources can be highly similar at the core genome level^15,16,28^. To characterise the relationship between ST131 isolates from Patient #1 and clinical ST131 isolates from the surrounding area, we compared their genomes with 25 contemporaneous Utah ST131 isolates and an international collection of 186 *E. coli* ST131 isolates from a recent large-scale evolutionary analysis^18^. Our phylogenetic analysis with 4219 non-recombinant core single-nucleotide polymorphisms (SNPs) revealed that Patient #1 isolates (U12A, U13A, U14A, U15A and U15B) form a discrete sub-clade within the previously defined ST131 phylogenetic clade C1 and are distinct from other Utah strains (Fig. 1). All five UTI-associated ST131 strains from Patient #1 were highly similar at the genome level, differing by only 26 core genome SNPs (Fig. 2). The strains also contain mutations in *gyrA* (S83L and D87N) and *parC* (S80I and E84V) that are found in other clade C1 isolates and confer high-level resistance to fluoroquinolones. These results are consistent with a clonal relationship among the isolates and indicate that UTI episodes between 2012 and 2015 are most likely due to the resurgence and/or re-inoculation of the urinary tract with the same strain. However, the isolates from 2013 and 2014 cluster apart from isolates from 2012, 2015 and 2016, suggesting co-existence of at least two sub-lineages of this clonal ST131 population that is likely specific to Patient #1 (Fig. 1). The 25 other contemporaneous Utah strains were distributed throughout the broader ST131 phylogeny with representatives from clades B (*n* = 4), C1 (*n* = 13) and C2 (*n* = 8), highlighting ST131 diversity within a defined geographic area (Fig. 1).

### Genome variation among Patient #1 ST131 isolates

Differences in the genome content between the draft genomes of Patient #1 isolates suggested that there may be variability in their mobile genetic elements (MGEs). We therefore used Pacific Biosciences (PacBio) single molecule real-time (SMRT) sequencing to obtain a complete genome sequence for representative Patient #1 isolates from each of the years 2012, 2013, 2014 and 2015 (U12A, U13A, U14A and U15A, respectively). We then determined the structure of all MGEs, including prophages, genomic islands (GIs) and plasmids. Whole-genome comparisons revealed the chromosome of all four isolates to be near identical, differing largely in their complement of insertion sequences (ISs) (Supplementary Table 3) and other MGEs (Supplementary Fig. 1a). Several MGEs and other discrete genomic loci are well conserved in ST131 clade C1^15,29,30^. As expected, the majority of these regions were similarly well conserved in Patient #1 isolates. However, some variation in the complement and composition of MGEs was evident, most notably an identical 42,657-bp deletion in the *leuX* GI of U12A and U15A, resulting in the loss of 50 putative protein-coding genes, including additional copies of the autotransporter gene *agn43* and the *fecABCD* iron transport operon (Supplementary Fig. 1b). We also identified a small deletion in a region containing genes encoding a type 6 secretion system in U14A and the complete loss of a genomic region encoding a chaperone-usher fimbriae locus (ECSF_4008-type)^31^ and a *picU*-like autotransporter gene in U15A, revealing a diverse range of structural genomic changes within these ST131 isolates.

### Plasmid loss among Patient #1 isolates

We observed substantial variation in plasmid content between Patient #1 ST131 genomes, including eight different circularised plasmid backbones, hereafter referred to by their designations in the U12A genome (Table 2; Supplementary Fig. 2). After screening the complete genome assemblies, draft Illumina assemblies and the associated long- and short-read sequence data, we found that only five plasmids were common to all Patient #1 isolates. These include four small, cryptic plasmids (pU12A-E, pU12A-F, pU12A-G, pU12A-H) ranging from 1.5 to 5.6 kb in size and a 69.5-kb IncFII plasmid (pU12A-C) that was highly similar (98% nucleotide sequence identity) to pC15-1a (GenBank accession: AY458016.1), a plasmid from an *E. coli* outbreak strain isolated from a long-term care facility in Toronto^32,33^. A second 151.7 kb IncF plasmid (pU12A-A) was identified in isolates collected prior to 2015 (U12A, U13A and U14A). pU12A-A is highly similar to the previously characterised *E. coli* plasmid pCA08 (GenBank accession: CP009233.1; 99% nucleotide sequence identity; 93% coverage) and carries a near identical complement of antimicrobial resistance genes^34^. U12A carried an additional 37.4 kb IncN3 plasmid, pU12A-D. pU12A-D is highly similar (BLASTn 95% nucleotide sequence identity, 89% coverage) to the *Klebsiella pneumoniae* plasmid pJIE137 (GenBank accession: EF219134.3) but lacks any resistance genes^35^.

**Table 2:** Plasmid distribution in P1A isolates

Plasmid name	Size (bp)	GC%	Inc. type	Resistance genes	U12A (MI-2)	U13A (MI-3)	U14A (MI-4)	U15A (MI-5)	U15B (MI-7)
pU12A-A	149,284	51.8	IncF (F1:A2:B20)	*aadA5*, *aac(3’)-Iid strA*, *strB*, *bla*_TEM-1B_ *mph(A)*, *sul1*, *sul2*, *tetA*, *dfrA17*	+	+	+	−	−
pU12A-B	87,359	50.33	IncI1	*None/aadA5*, *aac(3’)-Iid strA*, *strB*, *mph(A)*, *sul1*, *sul2*, *tetA*, *dfrA17*	+	−	−	+	−
pU12A-C	69,488	50.7	IncFII (F2:A-:B-)	None	+	+	+	+	+
pU12A-D	37,448	50.2	IncN3	None	+	−	−	−	−
pU12A-E	5210	48.5	NT	None	+	+	+	+	+
pU12A-F	4083	49.4	NT	None	+	+	+	+	+
pU12A-G	5629	47.38	NT	None	+	+	+	+	+
pU12A-H	1506	50.2	NT	None	+	+	+	+	+

*NT* Non-typable

U12A also carried an 87.3 kb IncI1 plasmid, pU12A-B, similar to IncI1 plasmids like the well-characterised R64 plasmids (GenBank accession: AP005147.1) that are often found in *Salmonella* species^36–38^. Plasmid pU12A-B was also identified in the genome of U15A but is not present in the genomes of the other urine isolates. Nearly identical IncI1 plasmids with similar resistance genes were identified in 24% (6/25) of the other contemporaneous Utah ST131 isolates (Supplementary Fig. 3). However, in these strains the IncI1 plasmid is missing a 3299-bp region (bases 51,594–54,863 in pU12A-B). Thus there appears to be an IncI1 plasmid backbone in circulation among otherwise genetically unrelated ST131 isolates in this geographic region that is near identical to the one observed in the Patient #1 ST131 isolates.

### Antibiotic resistance gene transfers in Patient #1

In silico resistance gene profiling of Patient #1 isolates revealed that the majority of acquired antibiotic resistance genes were located in a plasmid-borne 21.4 kb MDR island that is flanked and interspaced with five identical IS*6* ISs (Supplementary Fig. 4a). The MDR region is composed of a class 1 integron and several composite transposons that contain genes conferring resistance to trimethoprim, sulfonamides, macrolides, tetracycline and aminoglycosides (Table 2). In U12A, U13A and U14A, the MDR island is carried on the IncF plasmid pU12A-A, which also has the β-lactamase gene *bla*_TEM-1_. The MDR regions of all three isolates are identical with the exception of the gentamicin resistance transposon, which is notably absent in U13A and U14A. It is likely that this transposon has been lost through homologous recombination between IS*6* elements flanking the *aac(3)-IId* gene, deleting the transposon and leaving behind a single IS*6* element, as has been observed with other tandem arrays of IS-flanked resistance genes^39^. Although U15A lacks the IncF antibiotic resistance plasmid found in other isolates, in U15A the MDR region is carried on the IncI1 plasmid, pU12A-B. The presence of 8-bp direct target repeats flanking the MDR island indicate a recent transposition event, suggesting mobilisation and transfer of the entire island from the IncF plasmid pU12A-A to the IncI1 plasmid pU12A-B followed by the loss of pU12A-A (Supplementary Fig. 4b). The loss of pU12A-A in U15A also resulted in a change in its methylation profile, determined by exploiting the ability of PacBio SMRT sequencing to detect modified bases. Plasmid pU12A-A contains a type I methyltransferase (locus tag U12A_A0088) and its loss resulted in lack of modification of its predicted target sequence (5′-CCAGN_6_RTTG-3′) on the chromosome of U15A compared to U12A, U13A and U14A (Supplementary Table 4). Intriguingly, additional differences in the methylation profile were also observed between U15A and U12A, U13A and U14A, despite the fact that their complement of chromosomal methyltransferase genes is conserved (Supplementary Table 4).

Plasmids pU12A-A and pU12A-B were both absent in U15B, and susceptibility testing revealed it to be sensitive to nearly all antibiotics tested (Supplementary Table 2). In silico antibiotic resistance gene profiling of U15B showed that it lacked all of the plasmid-encoded antibiotic resistance genes identified in the other four Patient #1 isolates, suggesting the MDR island is completely absent in this strain (Table 1).

### Comparison of Patient #1 faecal and urine isolates

Our phylogenetic analysis of the Utah ST131 strains excluded a community reservoir as the likely source for the recurrent infection in Patient #1. Of note, the spouse of Patient #1 did not appear to be a carrier of the ST131 isolates based on urine and faecal analyses, and there were no household pets that might serve as carriers. Therefore, we hypothesised that the immediate reservoir of the urine isolates may be the intestinal tract of Patient #1. To test this hypothesis, we sequenced two faecal ST131 isolates collected from Patient #1 in 2015, choosing as representatives a trimethoprim-sensitive (TmS) isolate (F15sA) and a trimethoprim-resistant (TmR) isolate (F15rA). F15sA and F15rA were clonally related, with only four core SNPs, and both faecal isolates differed from U12A by fewer than eight core genome SNPs. Overall, the five urine and two faecal isolates from Patient #1 differ by only 27 uniquely mapping, non-recombinant core genome SNPs. Phylogenetically, the ST131 strains from Patient #1 can be separated into two closely related clusters: a small, diverse cluster comprised of the two urine isolates from 2013 and 2014 (U13A and U14A, respectively) and a dominant cluster containing the remaining urine isolates (U12A, U15A and U15B) and the two faecal isolates (F15sA and F15rA) (Fig. 2). Cumulatively, these results suggest that a population of ST131 were resident in the intestinal tract of Patient #1 where they may serve as a major reservoir for rUTIs.

### Antibiotic resistance heterogeneity among Patient#1 ST131

**Fig. 3:**
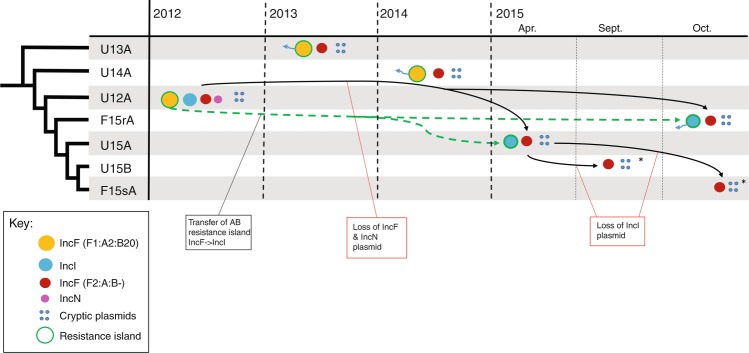
Plasmid dynamics in Patient #1 isolates. Isolates are listed in the first column in order of their phylogenetic relationship. The time line on the *x* axis indicates the approximate date of isolation of each strain. Black arrows indicate plasmid succession where directionality can be inferred from the single-nucleotide polymorphism profile of Patient #1 isolates (Fig. 2). Green arrows represent the transfer of the antibiotic resistance island from the multidrug resistance IncF plasmid to the IncI plasmid. Blue arrows extending from resistance plasmids represent the loss of the Gentamicin resistance transposon

**Fig. 4:**
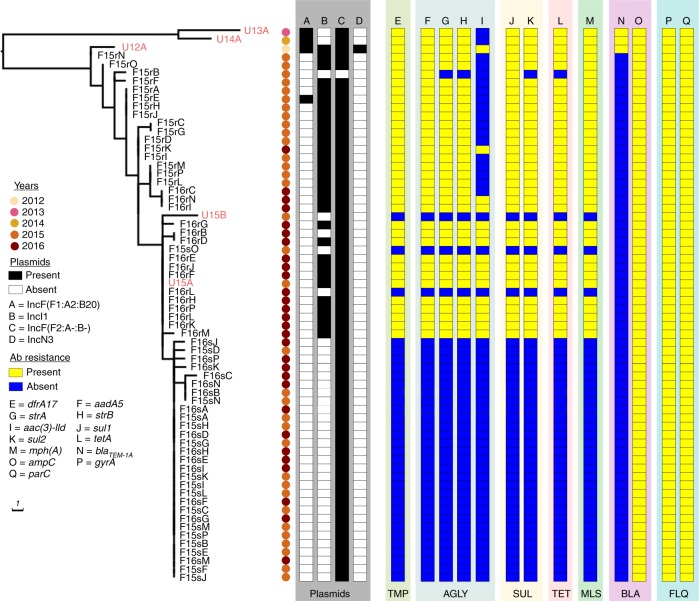
Phylogeny of the 62 Patient #1 urine and faecal isolates. The presence or absence of typeable plasmids and antibiotic resistance genes is indicated as per the legend. Plasmid’s presence or absence was determined based on a BLASTn comparison of the assembled contigs for each strain using the complete plasmid sequences from U12A as a reference (Supplementary Fig. 2). Scale bar indicates branch length in number of single-nucleotide polymorphisms. Antibiotic classes are abbreviated as follows: TMP trimethoprim, AGLY aminoglycoside, SUL sulfonamide, TET tetracycline, MLS macrolides, BLA beta-lactams, FLQ fluoroquinolones

As observed for the five urine ST131 isolates from Patient #1, the faecal isolates F15sA and F15rA exhibited heterogeneity in both plasmid and antibiotic resistance gene content. Specifically, F15rA carried the same MDR IncI plasmid as U15A, whereas F15sA was more similar to U15B in that both isolates lacked the MDR IncI plasmid (Fig. 3). Based on these observations, we speculated that both antibiotic-resistant and antibiotic-sensitive sub-populations of ST131 must co-exist in the gut microbiota of Patient #1. To obtain a clearer picture, we sequenced an additional 60 faecal isolates collected in 2015 and 2016. The faecal isolates were selected based on their sensitivity to trimethoprim, with 50% being TmS and 50% TmR. Resistance to trimethoprim is encoded as a component of the MDR island carried on plasmid pU12A-A or pU12A-B. Thus sensitivity/resistance to trimethoprim is a key indicator of MDR plasmid carriage among Patient #1 isolates. All 60 faecal ST131 isolates clustered in a single sub-lineage with U12A and the previously sequenced 2015 isolates (Fig. 4). A series of nested sub-clades were differentiated by one or two SNPs from their more ancestral isolates consistent with within-host micro-evolution (Fig. 4 and Supplementary Table 5). Trimethoprim resistance and susceptibility correlated with MDR region’s presence or absence, respectively (Fig. 4). In addition, TmR faecal isolates had an IncI plasmid consistent with the maintenance of the same MDR IncI plasmid that we completely sequenced from the U15A and F15rA isolates (Fig. 4; Supplementary Fig. 2). Absence of the gentamicin resistance transposon was observed in roughly half of the TmR faecal isolates, consistent with loss of this region from the MDR plasmid on at least two independent occasions (Fig. 4). In summary, all 2015/2016 isolates exhibited a high degree of sequence conservation, with only 24 SNPs defining the clade and no >3 SNPs separating any faecal isolates from at least 1 urine isolate (Supplementary Data 1). No further isolates similar to U13A and U14A were identified, suggesting that their sub-lineage may have been in a minority in 2015/2016 or became extinct (Fig. 4).

To better understand the plasmid dynamics within the gut population of Patient #1, we examined the relationship between the core genome phylogeny and the MDR IncI1 plasmid. Of the 30 TmS faecal isolates, 28 clustered in a terminal sub-clade with F15sA and 20 of these were genetically indistinguishable from F15sA at the core genome level (Fig. 4). The F15sA sub-clade is nested within a clade that includes seven TmR isolates and two TmS isolates that are genetically identical to U15A at the core SNP level (Fig. 4). These results indicate that the MDR IncI1 plasmid has been maintained in a sub-population of cells for at least 4 years (Fig. 1). All of the more ancestral isolates are TmR, suggesting that the loss of the IncI1 MDR plasmid from a sub-population of cells occurred relatively recently. Thus isolates that are genetically indistinguishable at the core genome level can be highly heterogeneous in terms of plasmid and antibiotic resistance gene content, suggesting that antibiotic resistance among ST131 bacteria within the intestinal microbiota of Patient #1 might be in a constant state of flux.

## Discussion

Here we used whole-genome sequencing to characterise ST131 strains causing rUTIs in a single individual (Patient #1) over a 5-year period. Our results provide compelling evidence that Patient #1 is persistently colonised by a cohort of very closely related ST131 strains that are able to cause frequent intermittent UTIs despite extensive exposure to multiple different antibiotics. The analysis of numerous faecal isolates indicates that antibiotic-sensitive and antibiotic-resistant variants of the ST131 strain co-exist within the intestinal tract of Patient #1. It is probable that these bacteria act as a major source for rUTI in Patient #1, in line with the common belief that rUTIs are often seeded by members of the host faecal microbiota^40,41^. However, our data do not rule out the existence of other reservoirs, which may persist at extraintestinal sites, such as within the bladder mucosa^42,43^.

Whole-genome sequencing is rapidly being adopted as a diagnostic tool for analysing clinical outbreaks where it accelerates pathogen identification, expedites the profiling of resistance and virulence genes and helps to rapidly pinpoint probable sources and likely vectors of disease. However, short-read sequencing data are ineffective at assembling large, complex plasmids, GIs and other repeat-dense genomic elements^44–46^. Here we used short-read Illumina sequencing to demonstrate the close genomic relationship between multiple ST131 isolates causing rUTI in a single patient and complemented this with long-read SMRT sequencing to generate high-quality, complete genome sequences of selected isolates that allowed us to: (i) fully characterise plasmid diversity within the resident ST131 population of Patient #1, (ii) completely resolve the structure and context of a large 21.7-kb MDR island, (iii) demonstrate the in vivo transfer of this island between plasmids with different evolutionary backgrounds, and (iv) identify differences in the methylome within this ST131 population.

We were interested to observe that all four small plasmids were assembled from the PacBio data alone in U14A, despite a seed length cut-off of 12 kb in HGAP^47^. Size selection during library preparation has been implicated in loss of small plasmids from PacBio assemblies^48,49^; however, we did not perform this step here. We reported small plasmid loss from an HGAP assembly when seed read cut-off was set higher than plasmid length^30^. However, this behaviour is unpredictable and it remains unclear why small plasmids could be assembled from U14A (but not others) in the present study. Consequently, for bacterial isolate sequencing we still recommend screening Illumina data for small plasmids in parallel with the assembly of corresponding PacBio data.

Interestingly, transfer of the MDR island coincides with loss of the original IncF plasmid pU12A-A, suggesting selective pressure to reduce the burden of carrying a large MDR plasmid during host adaptation. Loss of plasmid pU12A-A in U15A was also associated with changes in its epigenome; however, whether these changes have resulted in differential gene expression is currently unknown and requires further investigation. The diversity in methylome profiles within the resident ST131 population of Patient #1 could influence *E. coli* persistence in the gastrointestinal tract. Regardless of the mechanism(s) involved, the long-term persistence of both antibiotic-resistant and antibiotic-sensitive P1A lineage isolates suggests a population-level resistance strategy that allows sensitive cells to tolerate environmental antibiotic concentrations they could not normally survive.

Protective interactions between resistant and sensitive bacteria have been demonstrated under laboratory conditions^50–54^. For example, in a recent study it was shown that in a mixed population composed of ampicillin-resistant and ampicillin-sensitive *E. coli*, the sensitive isolates thrived in clinically relevant antibiotic concentrations 50 times greater than their minimal inhibitory concentrations (MICs)^51^. Similarly, in a co-culture of norfloxacin-resistant and norfloxacin-sensitive *E. coli*, the secretion of indole (a signalling molecule associated with stress tolerance) by resistant mutants enabled sensitive *E. coli* to grow in norfloxacin concentrations that they could not otherwise normally survive^54^. Cooperative resistance interactions between resistant and sensitive isolates could significantly increase fitness of the P1A lineage by enabling the population to rapidly adapt to fluctuating environmental antibiotic concentrations (such as those that might be encountered in an individual receiving treatment for rUTI). In this respect, long-term patient colonisation with carbapenem-resistant *K. pneumoniae* has also been examined using whole-genome sequencing, revealing extensive diversification of plasmid composition in the evolution of a clonal population^55^. However, to the best of our knowledge, this study represents a unique description of cooperative resistance in a patient suffering chronic rUTI.

Overall, our ability to characterise the structure and dynamics of an ST131 population in a single patient over a 5-year period (including monitoring changes in plasmid composition and antibiotic resistance elements) enhances our understanding of how bacteria respond to antibiotic treatment in patients who suffer chronic infection. Furthermore, the work provides a framework for the utilisation of precision medicine to manage and treat patients suffering debilitating chronic rUTI for which there is currently no effective cure.

## Methods

### Bacterial isolates

A collection of 92 *E. coli* strains, identified as ST131 by PCR^29,56,57^, was obtained between 2012 and 2016 from the University of Utah Urology Clinic and ARUP Laboratories. All strains were recovered from individuals residing in areas within and around Salt Lake City, UT. PCR was used to determine whether the strains encoded a *fimB* insertion^29^ or the O25b antigen^57^, both of which are often associated with ST131 strains. A third PCR used unique site-specific primers to identify and differentiate ST69, ST73, ST95 and ST131 lineages^56^. A list of all primers used in this study is provided in Supplementary Table 5. Sixty-seven of the 92 isolates were obtained from a single elderly female (Patient #1) with chronic rUTI. The remaining 25 isolates were recovered from urine samples that were selected randomly at the University of Utah Urology Clinic and ARUP Laboratories over a 7-month period in 2015, providing an indication of the local diversity of clinical ST131 isolates. A list of all isolates used in this study is provided in Supplementary Data 2.

The 67 Patient #1 isolates were obtained from both the urine (*n* = 5) and faeces (*n* = 62). A single clean batch urine isolate was obtained in each of the years 2012, 2013 and 2014 and a further two urine isolates were collected in 2015. Samples were plated on LB agar within a few hours post-collection and incubated overnight at 37 °C. Individual colonies were picked and grown in LB media for ~6 h prior to freezing in 15% glycerol for storage at −80 °C. For the 62 faecal isolates, 32 were obtained in 2015 and an additional 30 in 2016. Patient #1 had not received antibiotics for about 1.5 weeks prior to collection of the faecal samples. These samples were suspended and diluted in phosphate-buffered saline prior to plating on MacConkey agar ± ciprofloxacin (10 μg/mL) or trimethoprim (20 μg/mL). Faecal isolates from Patient #1 that were resistant to trimethoprim were also resistant to ciprofloxacin. To characterise the diversity in our antibiotic-resistant and antibiotic-sensitive sub-populations, equal numbers of resistant and sensitive faecal isolates were chosen for further analyses based on their sensitivity (2015, *n* = 16; 2016, *n* = 15) or resistance (2015, *n* = 16; 2016, *n* = 15) to trimethoprim (TmS and TmR, respectively).

### Antibiotic susceptibility testing

Antibiotic sensitivity test results and interpretations were generated using the NMIC-126 antimicrobial susceptibility testing panel on the Food and Drug Administration (FDA)-cleared BD Phoenix automated microbiology system (Becton Dickinson, Franklin Lakes, NJ). Susceptible, intermediate and resistant interpretations were derived by the system from MIC results using FDA-approved breakpoints.

### Genome sequencing and assembly

Genomic DNA extracted from all 92 isolates was sequenced using the Illumina NextSeq platform at the Australian Centre for Ecogenomics, University of Queensland. NextSeq DNA libraries were prepared using the Nextera XT library prep with Nextera XT indexes and sequenced using a 2 × 150 bp High Output V2 Kit. Raw Illumina sequencing data were quality filtered to remove Illumina adaptor sequences, low-quality bases (phred quality <5) and reads <80 bp. Quality-filtered Illumina reads were assembled using Spades v3.6.0 with default parameters.

Genomic DNA from four Patient #1 urine isolates (U12A, U13A, U14A and U15A) were sequenced using a Pacific Biosciences (PacBio) RS II without size selection (University of Malaya and the Translation Research Institute, UQ) with P6 polymerases and C4 sequencing chemistry. Sequence read data from a single SMRT cell for each sample was assembled de novo using the hierarchical genome assembly process (HGAP version 2) and quiver^47^ from the SMRT Analysis software suite (version 2.3.0—http://www.pacb.com/devnet/) with default parameters and seed read cut-off of 12 kb. Contigs generated during the assembly were visually screened for overlapping sequences on their 5′ and 3′ ends using Contiguity^58^. Overlapping ends, a characteristic feature of the HGAP assembly process, were manually trimmed based on sequence similarity and the contigs were circularised. Circularised contigs (chromosome and plasmids) were then subjected to a polishing phase, whereby the raw sequencing reads were mapped back onto the assembled circular contigs (BLASR and quiver^47,59^) to validate the assembly and improve overall sequence quality. An additional polishing step was required to resolve single-nucleotide insertion and deletions errors associated with homopolymer tracts. Illumina sequence data for U12A, U13A, U14A and U15A were aligned to their respective genomes using bwa version: 0.7.12-r1039^60^ and a corrected consensus was called using Pilon version 1.21 with default parameters and the ‘–fix indels’ flag^61^.

We detected 4 plasmids smaller than the seed read cut-off of 12 kb in the PacBio assembly of U14A. Together with the 4 large plasmids from the U12A PacBio assembly, these 8 plasmids were used as reference sequences to determine their distribution among the 67 Patient #1 isolates using BLASTn comparisons as implemented in BRIG^62^ (Supplementary Fig. 2).

### Genomic analysis with draft Illumina genomes

In silico multilocus sequencing typing (MLST) was carried out was carried out by read-mapping using SRST2 version 0.2.0^63^. Antibiotic resistance gene profiling was carried out using ResFinder version 2.1 and read-mapping approaches using Abricate version 0.2 (https://github.com/tseemann/abricate) and SRST2^63,64^. In silico plasmid incompatibility typing was carried out using PlasmidFinder version 1.3 and plasmid MLSTs were determined using pMLST version 1.4^65^.

To determine the phylogenetic relationship of ST131 isolates from this study in the global context, we carried out a phylogenomic analysis with 186 publicly available *E. coli* ST131 draft genomes collected from several countries^18^. Draft assemblies were aligned to the chromosome of the *E. coli* ST131 strain EC958 reference genome using parsnp^66^. Recombinant regions were filtered from the alignment using Gubbins v 2.1.0^67^ producing an alignment of 4286 core SNPs that have been vertically inherited. A maximum-likelihood tree was estimated using RAxML^68^ under the GTRGAMMA nucleotide substitution rate model.

To generate a maximum-likelihood tree of P1A isolates, trimmed reads were mapped to the complete chromosome sequence of U12A using Bowtie2. SNP calling and indel prediction were performed using Snippy version 2.9 (https://github.com/tseemann/snippy). Recombinant SNPs were removed using Gubbins producing an alignment of 39 core SNPs. A maximum-likelihood tree was estimated using RAxML under the GTRGAMMA nucleotide substitution rate model.

### Genome annotation and comparative analysis

Gene calling and automated functional annotation of the complete genomes of U12A, U13A, U14A and U15A were performed using Prokka and a custom *Escherichia* genus database^69^ followed by manual curation of ISs, integrons, prophage and restriction/modification systems with ISFinder, Integrall, Phaster and REBASE, respectively^70–73^. The Artemis Comparison Tool^74^, BRIG^62^ SeqFindR (https://github.com/mscook/SeqFindR) and EasyFig^75^ were used to visually compare the genomes of the 92 *E. coli* ST131 isolates identified in this study and to identify differences in plasmid content and other previously defined, conserved ST131 MGEs^15,29^.

### Ethics and consent

The research described in this study was undertaken with approval from the University of Utah Institutional Review Board (IRB_00070563). The patient provided both written and oral consent to participate in the study and to have her details published.

### Reporting summary

Further information on research design is available in the Nature Research Reporting Summary linked to this article.

## Supplementary information

Supplementary Information

Description of Additional Supplementary Files

Supplementary Data 1

Supplementary Data 2

Reporting Summary

## Data Availability

Genome data have been deposited to NCBI under the Bioprojects PRJNA516746 [https://www.ncbi.nlm.nih.gov//bioproject/516746], PRJNA516747 [https://www.ncbi.nlm.nih.gov//bioproject/516747], PRJNA516748 [https://www.ncbi.nlm.nih.gov//bioproject/516748], PRJNA516749 [https://www.ncbi.nlm.nih.gov//bioproject/516749] and PRJNA520966 [https://www.ncbi.nlm.nih.gov//bioproject/520966]. Raw PacBio sequence read data for isolates U12A, U13A, U14A and U15A have been deposited to the Sequence Read Archive (SRA) under the accession numbers SRR8504955, SRR8504956, SRR8504974 and SRR8509633. Illumina sequence read data for all 92 isolates have been deposited to the SRA under the accession numbers SRR8541965–SRR8542056. The complete genomes of U12A, U13A, U14A and U15A have been deposited to GenBank (accession numbers: U12A=CP035468–CP035476; U13A=CP035477–CP035483; U14A=CP035516–CP035522; U15A=CP035720–CP035726).
