# Supplementary material for: Population dynamics of an Escherichia coli ST131 lineage during recurrent urinary tract infection
Source: Nat Commun. 2019 Aug 13;10:3643. doi: 10.1038/s41467-019-11571-5 (PMC6692316; doi:10.1038/s41467-019-11571-5)
Supplement: Supplementary file 1 — Supplementary Information [file 41467_2019_11571_MOESM1_ESM.pdf]

Supplementary Information

Population dynamics of an *Escherichia coli* ST131 lineage during recurrent urinary tract infection

Brian M. Forde[1,2,3], Leah W. Roberts[1,2,3], Minh-Duy Phan[1,3], Kate M. Peters[1,3], Brittany A. Fleming[4], Colin W. Russell[4], Sara M. Lenherr[5], Jeremy B. Myers[5], Adam P. Barker[6], Mark A. Fisher[6], Teik-Min Chong[7], Wai-Fong Yin[7], Kok-Gan Chan[7,8], Mark A. Schembri[1,3*], Matthew A. Mulvey[4*] and Scott A. Beatson[1,2,3*]

[1]School of Chemistry and Molecular Biosciences, The University of Queensland, Brisbane, Queensland, Australia.

[2]Australian Infectious Diseases Research Centre, The University of Queensland, Brisbane, Queensland, Australia.

[3]Australian Centre for Ecogenomics, The University of Queensland, Brisbane, Queensland, Australia.

[4]University of Utah School of Medicine, Department of Pathology, Division of Microbiology and Immunology, Salt Lake City, Utah, USA.

[5]Genitourinary Injury and Reconstructive Urology, Department of Surgery, University of Utah, Salt Lake City, Utah, USA.

[6]ARUP Laboratories and Department of Pathology, University of Utah, Salt Lake City, Utah, USA.

[7]Division of Genetics and Molecular Biology, Institute of Biological Sciences, Faculty of Science, University of Malaya, Kuala Lumpur, Malaysia.

[8]International Genome Centre, Jiangsu University, Zhenjiang, China

* To whom correspondence should be addressed: A/Prof Scott Beatson (s.beatson@uq.edu.au), Prof Matthew Mulvey (mulvey@path.utah.edu) and Prof Mark Schembri (m.schembri@uq.edu.au).

Supplementary Methods

Detection of DNA modifications

The detection of epigenetic signatures was carried out using the RS_Modification and Motif Analysis.1 tool from the SMRT analysis package version 2.3.0. In brief, PacBio reads were mapped to the genomes of U12A, U13A, U14A and U15A. Interpulse durations (IPDs) [1] for each base were measured against an *in silico* kinetic reference model to determine their methylation status (http://www.pacb.com/wp-content/uploads/2015/09/WP_Detecting_DNA_Base_Modifications_Using_SMRT_Sequencing.pdf). Sequence motifs were identified using Motif finder v1 with default parameters.

Supplementary Table 1: Patient #1 prescribed antibiotics 2010-2016

Date	Antibiotic	Dosage
Dec 2010	Ceftriaxone	–
Jan 2011	Pyrazinamide	1 g 4X per day for 14 days
Feb 2011	Augmentin	500 mg Po BID for 14 days
Mar 2011	Cefedime	500 mg PO BID for 14 days
May 2011	Macrobid	100 mg for 14 days
Jun 2011	Zyvox	600 mg for 14 days, every 12 hrs
Jul 2011	Macrobid	100 mg for 28 days
Oct 2011	Nitrofuantoin/Macrobid	–
Apr 2012	Ertapenum	PICC line, 21 days; patient was free of UTI for ~9 months
Mar 2013	Nitrofuantoin	100 mg
Jul 2013	Ertapenem	–
Sept 2013	Ertapenem	7 days
Sept 2013	Levaquin	7 days
Sept 2013	Meropenem	–
Sept 2013	Cefepime	–
Sept 2013	Meropenem	1 g
Nov 2013	Zyvox	600 mg for 10 days
Nov 2013	Levaquin	750 mg for 14 days
Nov 2013	Amoxicillin	500 mg for 7 days
Nov 2013	Recephin	1 g for 2 days
Nov 2013	Rosephin	14 days
Nov 2013	Amoxicillin	500 mg for 10 days
Jan 2014	Ceftriaxone	–
Jan 2014	Nitrofurantoin	100 mg for 7 days
Mar 2014	Ciprofloxacin	500 mg
Apr 2014	Ciprofloxacin	10 days
Oct 2014	Ertapenem	1 g for 14 days
Feb 2015	Bactrim DS	–
Mar 2015	Ciprofloxacin	500 mg for 14 days
Apr 2015	Nitrofurantoin	100 mg
Apr 2015	Ertapenem	1 g for 10 days

May 2016	Levaquin	–
Jun 2016	Macrobid	–
Nov 2016	Macrobid	100 mg for 10 days

Supplementary Table 2: Antibiograms of U12A, U13A, U14A, U15A and U15B

Strain	Amikacin	Amoxicillin-Clavulanate	Ampicillin	Aztreonam	Cefazolin	Cefepime	Ceftazidime	Ceftriaxone	Cefuroxime	Ciprofloxacin	Gentamicin	Imipenem	Levofloxacin	Meropenem	Moxifloxacin	Nitrofurantoin	Piperacillin-Tazobactam	Tobramycin	Trimethoprim-Sulfamethoxazole
U12A	S	I	R	S	R	S	S	S	S	R	R	S	R	S	R	S	S	R	R
U13A	S	S	R	S	S	S	S	S	S	R	S	S	R	S	R	S	S	S	R
U14A	S	S	R	S	R	S	S	S	S	R	S	S	R	S	R	R	S	S	R
U15A	S	S	S	S	S	S	S	S	S	R	R	S	R	S	R	S	S	I	R
U15B	S	S	S	S	S	S	S	S	S	R	S	S	R	S	R	S	S	S	S

Susceptible (S)
Intermediate (I)
Resistant (R)

Supplementary Table 3: Comparison of IS insertions in the genomes of U12A, U13A, U14A and U15A

Ref*	Start	Stop	IS	U12A	U13A	U14A	U15A
U13A	685666	686418	IS1				
U13A	1196187	1196956	IS1				
U13A	1358046	1360577	ISEc23				
U13A	1372391	1372857	IS21-family-p				
U13A	1373334	1374564	ISEc24				
U13A	1418388	1419667	IS4-p				
U13A	1481006	1483586	ISEc12				
U13A	1533395	1534187	IS1				
U13A	1648576	1649867	ISEc1-p				
U13A	1786973	1787630	IS1				
U13A	1998603	2000020	IS1397				
U13A	2104805	2105756	IS609-p				
U13A	2136388	2137819	IS1397				
U13A	2192101	2194632	ISEc23				
U13A	2206314	2208035	ISEc38				
U13A	2211127	2212586	ISEc20				
U13A	2214613	2216475	IS100kyp-p				
U13A	2230816	2233396	ISEc12				
U13A	2244549	2245257	IS200C				
U13A	2558656	2559376	IS1				
U13A	2714166	2714934	IS1				
U13A	3071604	3074184	ISEc12				
U13A	3140393	3141642	ISEc52				
U13A	3248021	3248921	IS1				
U13A	3249129	3249896	IS1				
U13A	3251156	3251923	IS1				
U13A	3254146	3255455	IS1203				
U13A	3256113	3256887	ISL3-family-p				
U13A	3273826	3274152	IS600-p				
U13A	3274153	3275373	IS30				
U13A	3275377	3276312	IS600-p				
U13A	3276922	3279442	ISEc23				
U13A	3287228	3288554	IS2				
U13A	3298087	3300079	IS682-p				
U13A	3300080	3301651	IS66-family-p				
U13A	3334200	3335091	IS1				
U13A	3337771	3338539	IS1				
U13A	3497924	3499173	ISEc52				
U13A	3759872	3761329	IS609-p				
U13A	4150036	4151134	ISEc52				
U13A	4670901	4671684	IS1				
U13A	4684448	4684826	IS3-family-p				
U13A	4709093	4710350	IS3				
U13A	4713399	4713616	IS66-family-p				
U13A	4713617	4714533	IS3-family-p				
U13A	4714534	4714850	IS3-family-p				
U13A	4715812	4716576	ISEc30				
U13A	4722377	4723152	IS1				

U13A	4725107	4725878	IS1
U13A	4726455	4727226	IS1
U13A	4867810	4868080	IS66-family-p
U13A	4874941	4875256	IS3-family-p
U13A	4877094	4879625	ISEc23
U13A	4890109	4891373	IS600
U13A	4891376	4892164	ISEc52-p
U13A	4900839	4901606	IS1
U13A	4903724	4906012	ISEc8-p
U13A	4917363	4919943	ISEc12
U13A	4923252	4925772	ISEc23
U13A	4926930	4927545	IS3-family-p
U13A	4937421	4938670	ISEc52
U13A	4958850	4960099	ISEc52
U13A	4966319	4968203	ISEc55

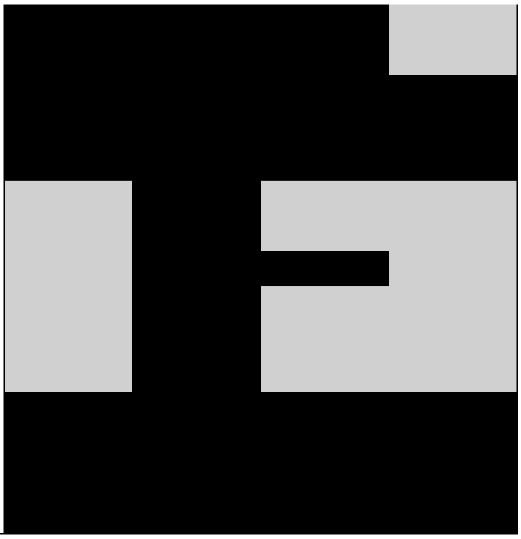

*IS coordinates are relative to the genome of U13A.

Present

Absent

Supplementary Table 4: Summary of methyltransferase genes and R-M systems in the genomes of U12A, U13A, U14A and U15A[##]

Strain	Locus tag	Type	Mod Type	Recognition motif	% mod	nDetected	nGenome
U12A	U12A_04762	Type I	m6A	AACN$_4$CTTT/AAAGN$_4$GTT	100/100	893/893	893/893
	U12A_00296	Type I	m6A	CACN$_4$GTAY/RTACN$_4$GTG**	99.8/99.7	740/739	741/741
	U12A_A0088	Type I	m6A	CCAGN$_6$RTTG/CAAYN$_6$CTGG*[#]	99.7/98.1	846/832	848/848
	U12A_04693	Type IIS	m6A	GAGACC*	99.2	422	425
	U12A_03616	Dam	m6A	GATC	99	42126	42530
U13A	U13A_04807	Type I	m6A	AAAGN$_4$GTT/AACN$_4$CTTT	100/100	872/872	872/872
	U13A_00295	Type I	m6A	CACN$_4$GTAY/RTACN$_4$GTG**	100/99.8	727/726	727/727
	U13A_A0085	Type I	m6A	CCAGN$_6$RTTG/CAAYN$_6$CTGG*	99.5/98	830/818	834/834
	U13A_03611	Dam	m6A	GATC	99	41476	41872
	U13A_04738	Type IIS	m6A	GAGACC	99	403	407
U14A	U14A_04810	Type I	m6A	AACN$_4$CTTT/AAAGN$_4$GTT	100/99.8	872/871	872/872
	U14A_00296	Type I	m6A	CACN$_4$GTAY/RTACN$_4$GTG**	100/99.8	727/726	727/727
	U14A_A0089	Type I	m6A	CCAGN$_6$RTTG/CAAYN$_6$CTGG*	99.5/97.7	830/815	834/834
	U14A_03614	Dam	m6A	GATC	99	41458	41872
	U14A_04741	Type IIS	m6A	GAGACC*	98.7	402	407
U15A	U15A_04671	Type I	m6A	AAAGN$_4$GTT/AACN$_4$CTTT	99.8/99.2	854/849	855/855
	U15A_00295	Type I	m6A	CACN$_4$GTAY **	99.4/na	697/na	701/na
	U15A_03610	Dam	m6A	GATC	98.5	40474	41054

* Absent in U15A; ** hemi-methylated in U15A; [#] CCAGN$_6$RTTG/CAAYN$_6$CTGG is characteristic of a type I recognition motif. Loss of the type I R-M system U12A_A0088 in U14A, allowed us to determine its recognition motif.[##] All four genomes were methylated at 5'-G^{m6}ATC-3' deoxyadenosine methylase (Dam) motifs, the frequency of which was consistent with other *E. coli* methylomes [2,3]. U12A, U13A and U14A carry active homologs of the EC958 type I restriction-modification (R-M) systems EcoMIII and EcoMII that target AACN$_4$CTTT and CACN$_4$GTAY, respectively [2], and an active homolog of the type II R-M system Eco31I that targets 5'-GAG^{m6}ACC-3' motifs [4]. U12A, U13A and U14A also possess an undefined type I MTase (locus tag U12A_A0088) that is carried on the IncF plasmid pU12A-A and, based on sequence homology (BLASTn versus REBASE [5]), is predicted to target 5'-CCAGN$_6$RTTG-3' motifs. In the case of U15A, despite carrying homologs of Eco31I and EcoMII, we observed no methylation of 5'-GAG^{m6}ACC-3' motifs and only hemi-methylation of 5'-CACN$_4$GTAY-3' motifs, suggesting that the expression of these MTases is altered in this isolate. No methylation was detected at 5'-CCAGN$_6$RTTG-3' sites throughout the genome of U15A, consistent with the absence of the type I RM system carried by plasmid pU12A-A that is present in U12A, U13A and U14A.

Supplementary Table 5: Primers used in this study

Target	Sequence	Reference
***fimB* insertion**		
fimB-IS_F	5'-TCCTGACCCATAGTGAAATCG	6
fimB-IS_R	5'-GCTCTATCCCAGATGCCGTA	6
***pabB* gene**		
O25pabBspe.F	5'-TCCAGCAGGTGCTGGATCGT	7
O25pabBspe.R	5'-GCGAAATTTTTCGCCGTACTGT	7
**ST-specific primers**		
ST73_for	5'-TGGTTTTACCATTTTGTCGGA	8
ST73_rev	5'-GGAAATCGTTGATGTTGGCT	8
ST131_for	5'-GACTGCATTTCGTCGCCATA	8
ST131_rev	5'-CCGGCGGCATCATAATGAAA	8
ST95_for	5'-ACTAATCAGGATGGCGAGAC	8
ST95_rev	5'-ATCACGCCCATTAATCCAGT	8
ST69_for	5'-ATCTGGAGGCAACAAGCATA	8
ST69_rev	5'-AGAGAAAGGGCGTTCAGAAT	8

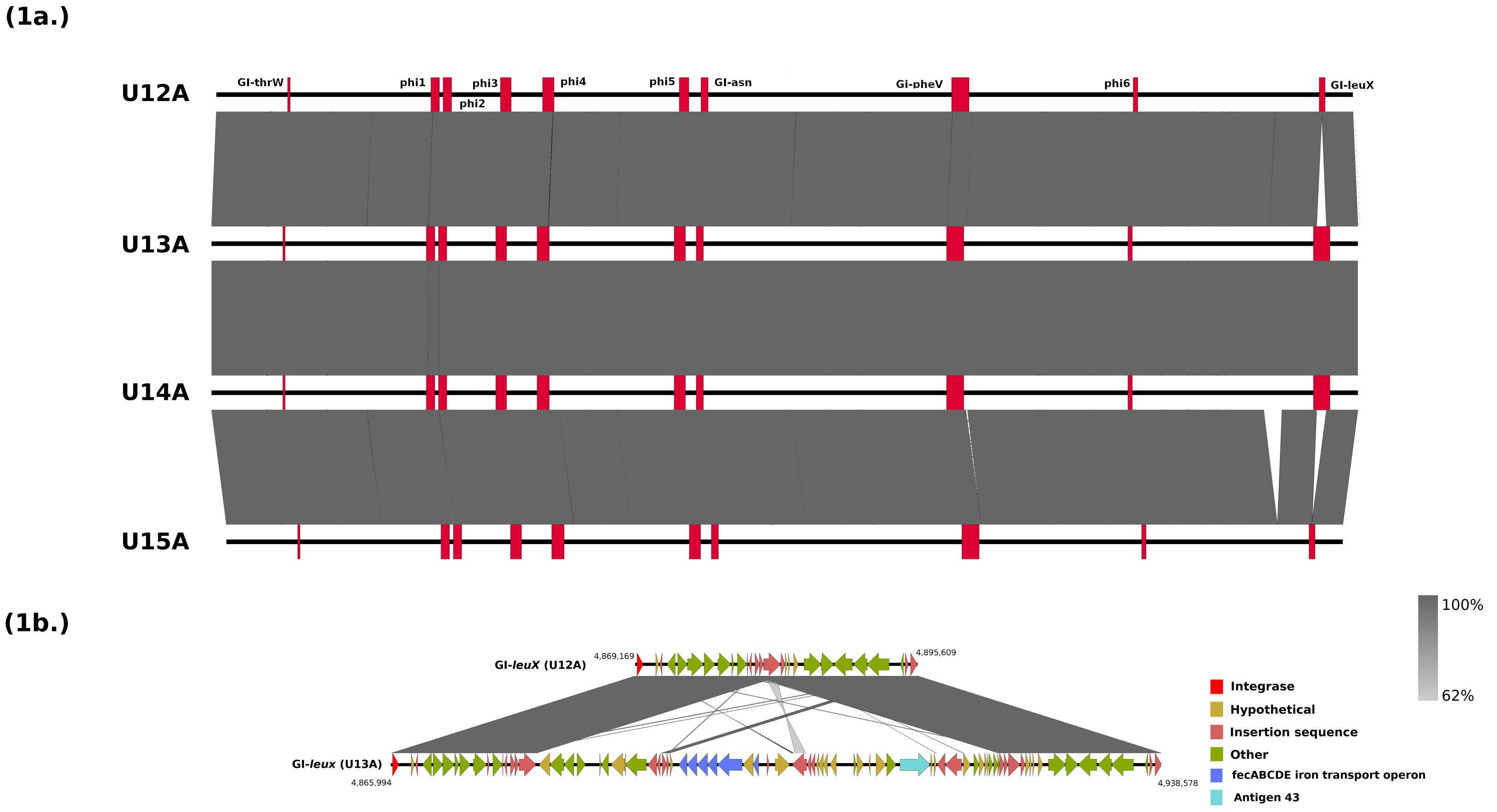

Supplementary Figure 1: (1a) Pairwise whole genome nucleotide comparisons (BLASTn) of 4 Patient #1 urine isolates highlighting differences in there MGEs. The large GI-*leuX* deletion is evident in U12A and U15A as is a deletion specific to U15A. **(1b)** Pairwise nucleotide comparison (BLASTn) of GI-leuX from U12A and U13A. Nucleotide comparisons were visualised using Easyfig.

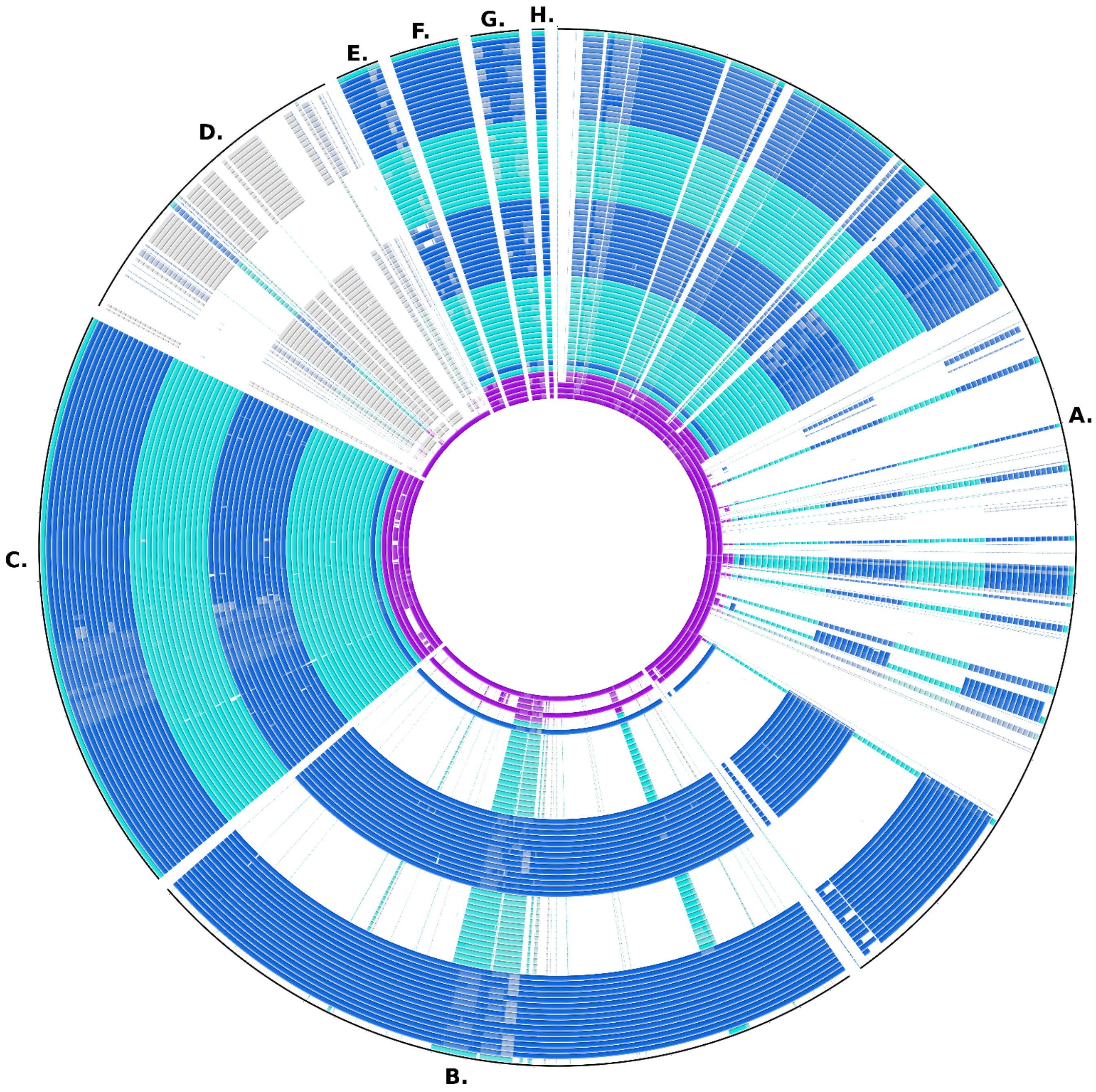

Supplementary Figure 2: BRIG visualisation showing the distribution of the 8 U12A plasmids among the Patient #1 isolates. Each circle displays BLASTn searches against each of the eight U12A plasmids which are labelled as follows: A=pU12A-A, B=pU12A-B, C=pU12A-C, D=pU12A-D, E=pU12A-E, F=pU12A-F, G=pU12A-G, H=pU12A-H. Patient #1 urine isolates U12A, U13A, U14A, U15A and U15B are arranged in the inner 5 purple rings (inner to outer, respectively). Patient #1 fecal isolates are arranged in the same order as Supplementary Table 6. Trimethoprim resistant (TmR) fecal isolates are coloured in blue and trimethoprim sensitive (TmS) fecal isolates in teal.

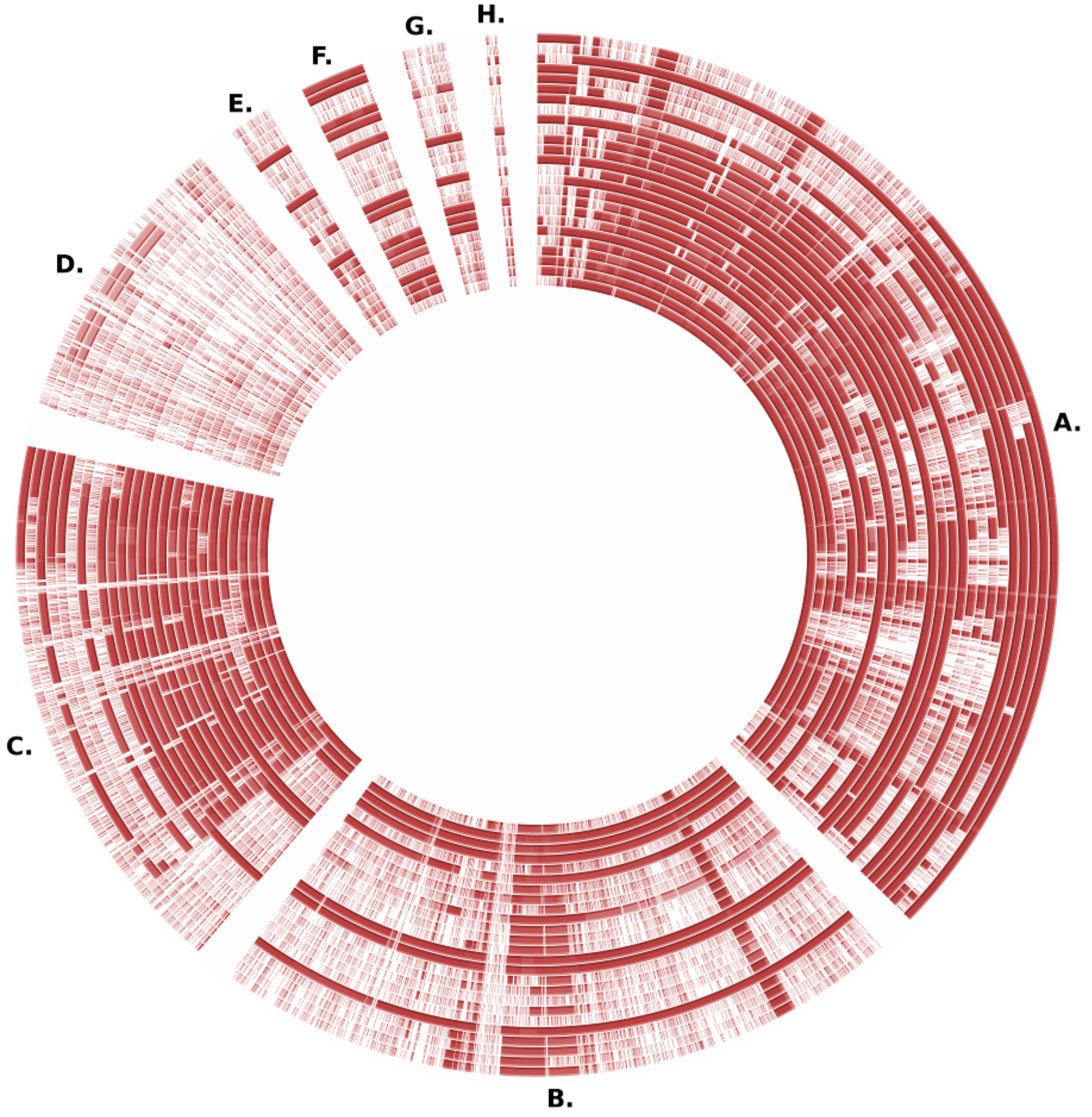

Supplementary Figure 3: BRIG visualisation showing the distribution of 8 U12A plasmids among the other ST131 isolates identified in this study. Each circle displays BLASTn searches against each of the eight U12A plasmids which are labelled as follows: A=pU12A-A, B=pU12A-B, C=pU12A-C, D=pU12A-D, E=pU12A-E, F=pU12A-F, G=pU12A-G, H=pU12A-H. Isolates are arranged in the same order as Supplementary Table 6.

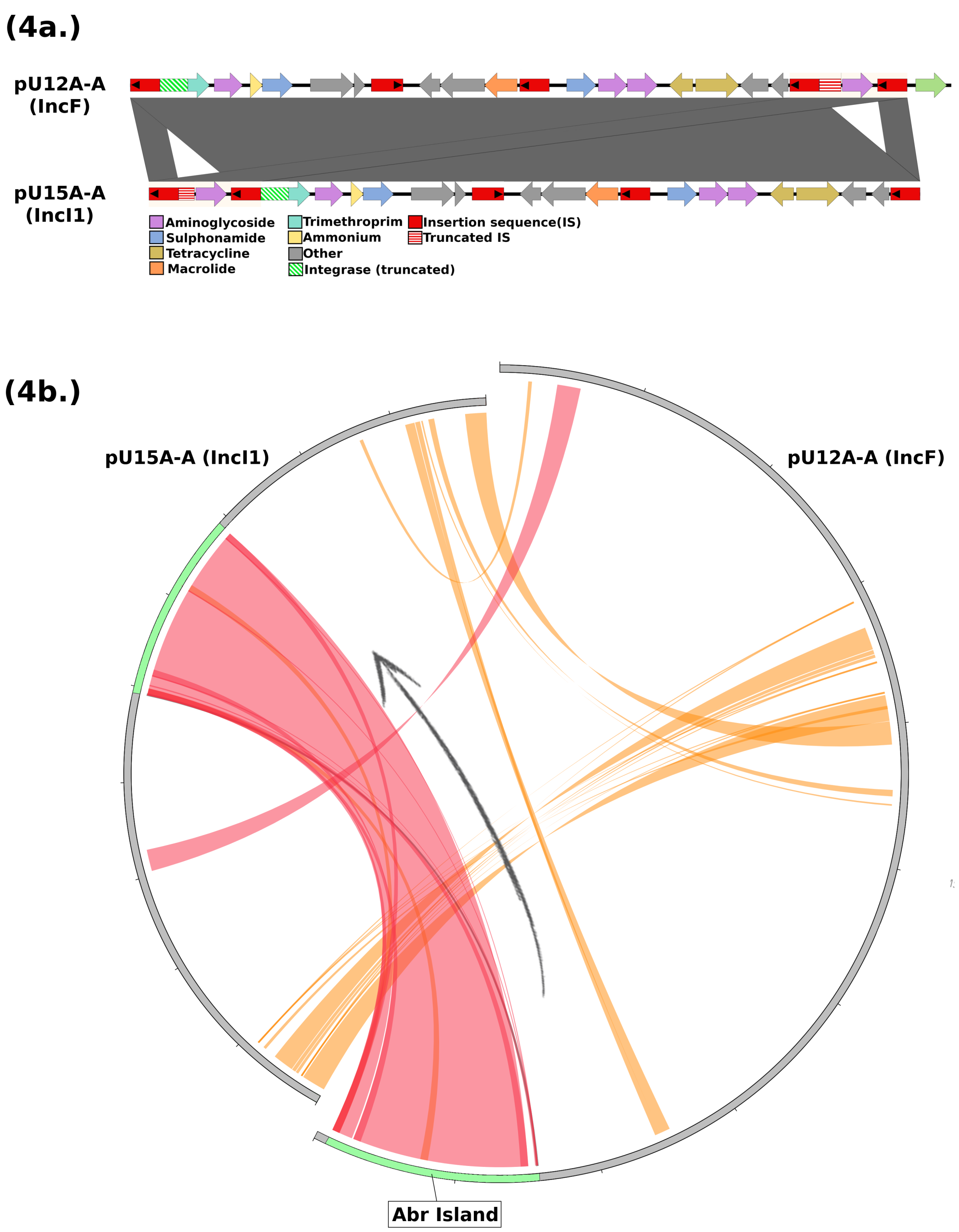

Supplementary Figure 4: (4a) Pairwise nucleotide comparison of antibiotic resistance islands from pU12A-A and pU15A-B. **(4b)** Pairwise whole genome nucleotide comparison (BLASTn) of the IncF plasmid pU12A-A from U12A and the IncI plasmid pU15A-B. The red ribbon represents a region of high sequence homology (>95% nucleotide sequence identity) corresponding to the resistance gene island (green) carried on pU12A-A. The arrow indicates the direction of transfer of the island from pU12A-A to pU15A-B.

Supplementary References

1 Flusberg, B. A. *et al.* Direct detection of DNA methylation during single-molecule, real-time sequencing. *Nat Methods* **7**, 461-465, doi:10.1038/nmeth.1459 (2010).

2 Forde, B. M. *et al.* Lineage-Specific Methyltransferases Define the Methylome of the Globally Disseminated Escherichia coli ST131 Clone. *MBio* **6**, e01602-01615, doi:10.1128/mBio.01602-15 (2015).

3 Fang, G. *et al.* Genome-wide mapping of methylated adenine residues in pathogenic Escherichia coli using single-molecule real-time sequencing. *Nature biotechnology* **30**, 1232-1239, doi:10.1038/nbt.2432 (2012).

4 Bitinaite, J. *et al.* Alw26I, Eco31I and Esp3I--type IIs methyltransferases modifying cytosine and adenine in complementary strands of the target DNA. *Nucleic Acids Res* **20**, 4981-4985 (1992).

5 Roberts, R. J., Vincze, T., Posfai, J. & Macelis, D. REBASE--restriction enzymes and DNA methyltransferases. *Nucleic Acids Res* **33**, D230-232, doi:10.1093/nar/gki029 (2005).

6 Totsika, M. *et al.* Insights into a multidrug resistant *Escherichia coli* pathogen of the globally disseminated ST131 lineage: genome analysis and virulence mechanisms. *PLoS One* **6** (2011).

7 Clermont, O. *et al*. Rapid detection of the O25b-ST131 clone of *Escherichia coli* encompassing the CTX-M-15-producing strains. *Journal of Antimicrobial Chemotherapy* **64**, 274-277 (2009).

8 Doumith, M. *et al.* Rapid identification of major *Escherichia coli* sequence types causing urinary tract and bloodstream infections. *Journal of Clinical Microbiology* **53**, 160-166 (2015).
