# Supplementary material for: Population dynamics of an Escherichia coli ST131 lineage during recurrent urinary tract infection
Source: Nat Commun. 2019 Aug 13;10:3643. doi: 10.1038/s41467-019-11571-5 (PMC6692316; doi:10.1038/s41467-019-11571-5)
Supplement: Supplementary file 2 — Description of Additional Supplementary Files [file 41467_2019_11571_MOESM2_ESM.pdf]

Description of Additional Supplementary Files

File Name: Supplementary Data 1

Description: Core genome SNPs of P#1 isolates relative to the genome of E. coli U12A

File Name: Supplementary Data 2

Description: Description of clinical isolates used in this study.
