# Supplementary material for: Population dynamics of an Escherichia coli ST131 lineage during recurrent urinary tract infection
Source: Nat Commun. 2019 Aug 13;10:3643. doi: 10.1038/s41467-019-11571-5 (PMC6692316; doi:10.1038/s41467-019-11571-5)
Supplement: Supplementary file 5 — Reporting Summary [file 41467_2019_11571_MOESM5_ESM.pdf]

nature research

Corresponding author(s): Mark A. Schembri, Matthew A. Mulvey, Scott A. Beatson

Last updated by author(s): Jul 16, 2019

Reporting Summary

Nature Research wishes to improve the reproducibility of the work that we publish. This form provides structure for consistency and transparency in reporting. For further information on Nature Research policies, see Authors & Referees and the Editorial Policy Checklist.

Statistics

For all statistical analyses, confirm that the following items are present in the figure legend, table legend, main text, or Methods section.

n/a	Confirmed	
☒	☐	The exact sample size (*n*) for each experimental group/condition, given as a discrete number and unit of measurement
☒	☐	A statement on whether measurements were taken from distinct samples or whether the same sample was measured repeatedly
☒	☐	The statistical test(s) used AND whether they are one- or two-sided<br>*Only common tests should be described solely by name; describe more complex techniques in the Methods section.*
☒	☐	A description of all covariates tested
☒	☐	A description of any assumptions or corrections, such as tests of normality and adjustment for multiple comparisons
☒	☐	A full description of the statistical parameters including central tendency (e.g. means) or other basic estimates (e.g. regression coefficient) AND variation (e.g. standard deviation) or associated estimates of uncertainty (e.g. confidence intervals)
☒	☐	For null hypothesis testing, the test statistic (e.g. *F*, *t*, *r*) with confidence intervals, effect sizes, degrees of freedom and *P* value noted<br>*Give P values as exact values whenever suitable.*
☒	☐	For Bayesian analysis, information on the choice of priors and Markov chain Monte Carlo settings
☒	☐	For hierarchical and complex designs, identification of the appropriate level for tests and full reporting of outcomes
☒	☐	Estimates of effect sizes (e.g. Cohen's *d*, Pearson's *r*), indicating how they were calculated

Our web collection on statistics for biologists contains articles on many of the points above.

Software and code

Policy information about availability of computer code

Data collection	*Provide a description of all commercial, open source and custom code used to collect the data in this study, specifying the version used OR state that no software was used.*
Data analysis	*Provide a description of all commercial, open source and custom code used to analyse the data in this study, specifying the version used OR state that no software was used.*

For manuscripts utilizing custom algorithms or software that are central to the research but not yet described in published literature, software must be made available to editors/reviewers. We strongly encourage code deposition in a community repository (e.g. GitHub). See the Nature Research guidelines for submitting code & software for further information.

Data

Policy information about availability of data

All manuscripts must include a data availability statement. This statement should provide the following information, where applicable:

- Accession codes, unique identifiers, or web links for publicly available datasets
- A list of figures that have associated raw data
- A description of any restrictions on data availability

Genome data has been deposited to NCBI under the Bioprojects PRJNA516746 [https://www.ncbi.nlm.nih.gov//bioproject/516746], PRJNA516747 [https://www.ncbi.nlm.nih.gov//bioproject/516747], PRJNA516748 [https://www.ncbi.nlm.nih.gov//bioproject/516748], PRJNA516749 [https://www.ncbi.nlm.nih.gov//bioproject/516749] and PRJNA520966 [https://www.ncbi.nlm.nih.gov//bioproject/520966]. Raw PacBio sequence read data for isolates U12A, U13A, U14A and U15A has been deposited to the Sequence Read Archive (SRA) under the accessions SRR8504955 [https://trace.ncbi.nlm.nih.gov/Traces/sra/?run=SRR8504955], SRR8504956 [[https://trace.ncbi.nlm.nih.gov/Traces/sra/?run=SRR8504956], SRR8504974 [[https://trace.ncbi.nlm.nih.gov/Traces/sra/?run=SRR8504974] and SRR8509633 [https://trace.ncbi.nlm.nih.gov/Traces/sra/?run=SRR8509633]. Illumina sequence read data for all 92 isolates has been deposited to the SRA under the accessions SRR8541965-SRR8542056 [https://www.ncbi.nlm.nih.gov/sra?linkname=bioproject_sra_all&from_uid=520966]. The complete genomes of U12A, U13A, U14A and U15A have been deposited to GenBank (accessions: U12A=CP035468-CP035476 [https://www.ncbi.nlm.nih.gov/nuccore?term=516746%5BBioProject%5D]; U13A=CP035477-CP035483 [https://www.ncbi.nlm.nih.gov/nuccore?term=516747%5BBioProject%5D]; U14A=CP035516-CP035522 [https://

www.ncbi.nlm.nih.gov/nuccore?term=516748%5BBioProject%5D]; U15A=CP035720-CP035726 [https://www.ncbi.nlm.nih.gov/nuccore?term=516749%5BBioProject%5D]).

Field-specific reporting

Please select the one below that is the best fit for your research. If you are not sure, read the appropriate sections before making your selection.

☒ Life sciences ☐ Behavioural & social sciences ☐ Ecological, evolutionary & environmental sciences

For a reference copy of the document with all sections, see nature.com/documents/nr-reporting-summary-flat.pdf

Life sciences study design

All studies must disclose on these points even when the disclosure is negative.

Sample size	Sample sizes were determined based on availability on isolates for sequence analysis
Data exclusions	No data were excluded from the analysis
Replication	This study involved the collection and genome sequencing of E. coli isolates from urine and fecal samples from a single patient over a 5-year period. It is not possible to repeat these experiments using a controlled protocol.
Randomization	Randomization was not relevant to this study.
Blinding	Blinding was not relevant to this study.

Reporting for specific materials, systems and methods

We require information from authors about some types of materials, experimental systems and methods used in many studies. Here, indicate whether each material, system or method listed is relevant to your study. If you are not sure if a list item applies to your research, read the appropriate section before selecting a response.

Materials & experimental systems

n/a	Involved in the study	
☒	☐	Antibodies
☒	☐	Eukaryotic cell lines
☒	☐	Palaeontology
☒	☐	Animals and other organisms
☐	☒	Human research participants
☒	☐	Clinical data

Methods

n/a	Involved in the study	
☒	☐	ChIP-seq
☒	☐	Flow cytometry
☒	☐	MRI-based neuroimaging

Human research participants

Policy information about studies involving human research participants

Population characteristics	The patient examined is a Caucasian female who first presented to the Urology Clinic at the University of Utah Hospital in 2011 at 74 years old. She has a pertinent past medical history of lifelong irritable bowel symptoms and colonic diverticulosis, and has been taking amitriptyline for nausea since the early 1980s. The patient also uses the ACE inhibitor Lisinopril (20 mg daily), the beta blocker Atenolol (50 mg daily), and the diuretic Hydrochlorothiazide (25 mg daily) to control high blood pressure and the non-steroidal anti-inflammatory drug Meloxicam (15 mg as needed) for back pain. She regularly takes vitamin pills, including iron supplements. The patient experiences frequent UTIs, which began soon after a vaginal hysterectomy in 1971. Her UTIs are accompanied by symptoms of dysuria and urinary frequency/urgency, fatigue, and bladder pain, all of which usually resolve with administration of culture-specific antibiotic treatments. In 2010, outside hospital providers became concerned about a possible colovesical fistula based on the patient's history of recurrent UTIs and the close proximity of her colon to the bladder, as revealed by CT scan imaging. The patient subsequently underwent resection of 12 inches of colon, and a flap of omentum was placed between the colon and bladder wall. This intervention did not reduce her UTI frequency. Over the years, the patient has suffered frequent UTIs and has been seen by multiple healthcare providers who have prescribed a variety of antibiotics.
Recruitment	The study involved the characterization of E. coli isolates recovered from the urine and feces of a single individual over a 5-year period
Ethics oversight	The research described in this study was undertaken with approval from the University of Utah Institutional Review Board (IRB_00070563). The patient provided both written and oral consent to participate in the study and to have her details published.

Note that full information on the approval of the study protocol must also be provided in the manuscript.
